# Synbiotic Effects of Fermented Rice on Human Health and Wellness: A Natural Beverage That Boosts Immunity

**DOI:** 10.3389/fmicb.2022.950913

**Published:** 2022-07-13

**Authors:** Shivkanya Fuloria, Jyoti Mehta, Manash Pratim Talukdar, Mahendran Sekar, Siew Hua Gan, Vetriselvan Subramaniyan, Nur Najihah Izzati Mat Rani, M. Yasmin Begum, Kumarappan Chidambaram, Rusli Nordin, Mohammad Nazmul Hasan Maziz, Kathiresan V. Sathasivam, Pei Teng Lum, Neeraj Kumar Fuloria

**Affiliations:** ^1^Faculty of Pharmacy, AIMST University, Bedong, Malaysia; ^2^Department of Applied Sciences and Biotechnology, Shoolini University, Solan, India; ^3^Department of Pharmaceutical Chemistry, Faculty of Pharmacy and Health Sciences, Royal College of Medicine Perak, Universiti Kuala Lumpur, Ipoh, Malaysia; ^4^School of Pharmacy, Monash University Malaysia, Bandar Sunway, Malaysia; ^5^Faculty of Medicine, Bioscience and Nursing, MAHSA University, Jenjarom, Malaysia; ^6^Faculty of Pharmacy and Health Sciences, Royal College of Medicine Perak, Universiti Kuala Lumpur, Ipoh, Malaysia; ^7^Department of Pharmaceutics, College of Pharmacy, King Khalid University, Abha, Saudi Arabia; ^8^Department of Pharmacology, College of Pharmacy, King Khalid University, Abha, Saudi Arabia; ^9^Faculty of Applied Sciences, AIMST University, Bedong, Malaysia; ^10^Centre for Transdisciplinary Research, Department of Pharmacology, Saveetha Dental College and Hospitals, Saveetha Institute of Medical and Technical Sciences, Saveetha University, Chennai, India

**Keywords:** fermented foods, rice beverage, *Xaj-pani*, starter culture, yeast, mold, lactic acid bacteria

## Abstract

Fermented foods have been an important component of the human diet from the time immemorial. It contains a high amount of probiotics that have been associated to a wide range of health benefits, including improved digestion and immunity. This review focuses on the indigenously prepared prebiotic- and probiotic-containing functional fermented rice (named *Xaj-pani*) by the Ahom Community from Assam, in Northeast India, including all the beneficial and potential effects on human health. Literature was searched from scientific databases such as PubMed, ScienceDirect and Google Scholar. Glutinous rice (commonly known as bora rice of sali variety) is primarily employed to prepare beverages that are recovered through the filtration process. The beer is normally consumed during religious rites, festivals and ritual practices, as well as being used as a refreshing healthy drink. Traditionally, it is prepared by incorporating a variety of medicinal herbs into their starter culture (*Xaj-pitha*) inoculum which is rich in yeasts, molds and lactic acid bacteria (LAB) and then incorporated in alcoholic beverage fermentation. The Ahom communities routinely consume this traditionally prepared alcoholic drink with no understanding of its quality and shelf life. Additionally, a finally produced dried cake, known as *vekur pitha* act as a source of *Saccharomyces cerevisiae* and can be stored for future use. Despite the rampant use in this community, the relationship between *Xaj-pani*’s consumption, immunological response, infectious and inflammatory processes remains unknown in the presence of factors unrelated or indirectly connected to immune function. Overall, this review provides the guidelines to promote the development of prebiotic- and probiotic-containing functional fermented rice that could significantly have an impact on the health of the consumers.

## Graphical Abstract

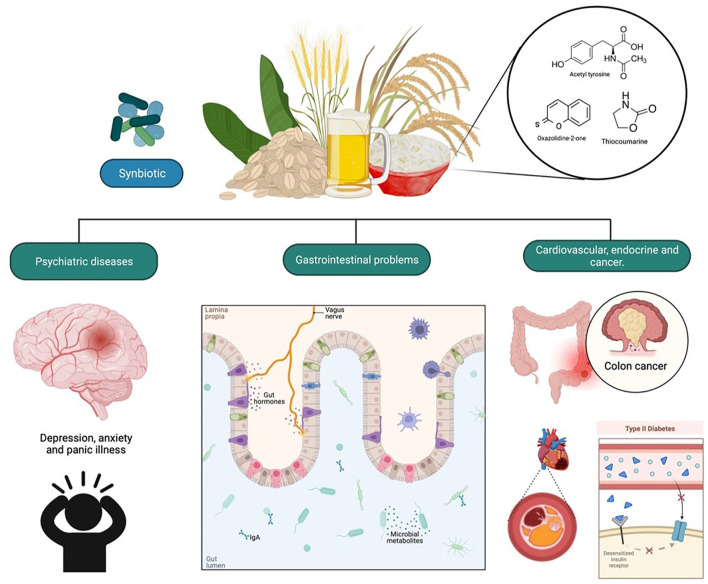

## Introduction

Fermented food is generally defined as an edible product produced from raw or cooked substances that are of plant or animal origin, produced by microorganisms either spontaneously or by adding in some cultures (Hansen, 2002). Fermentation is derived from the Latin word “fermentare,” meaning “to leaven.” It is the chemical breakdown process of a substance by yeasts, bacteria, or other microorganisms, typically involving an exothermic reaction. Fermented rice beverage is the most common alcoholic beverage of NE (NE) India; consumed by most of the native tribal communities, the inhabitant of the mountains as well as those at the Himalayan foothills. Globally, fermented food and beverages account for approximately one-third of the human diet. Traditionally prepared fermented food and rice-based alcoholic beverages like *Xaj-pani* or *Lao-pani* uses locally available raw materials and are being practiced even today by the descendants (Roy et al., 2004) especially in NE India comprising of Assam, Manipur, Arunachal Pradesh, Meghalaya, Mizoram, Nagaland, Tripura, and Sikkim. The North-Eastern part of India consisting of these eight states, has a majority of tribal communities that practices their own ethnic culture and background.

*Xaj-pani* is a variety of beer locally prepared from rice in Assam by the Ahoms. *Xaj-pani* or *Xaj* in short, is associated with the social and religious belief system of the *Ahoms* or *Tai*-*Ahoms*, an ethnic community of Assam (Saikia et al., 2007). It is consumed during the Bihu festival especially after conducting hard work/labor. In addition to *Ahom*s, some of the major tribal communities like *Mishing, Khamtis, Nagas*, and *Garos* are also known (Asada, 2012). A starter cake, known as “vekur-pitha,” which consists of various parts of several plant species like *Lygodium flaxuosum* Linn., *Cissampelos pareira, Scoparia dulcis* Linn., *Leucas aspera Spreng, Piper betle* Linn., and *Cinnamomum glanduliferum* Meissn is normally made prior to the fermentation and afterward added to the rice substrate for fermentation. The rice-based fermented alcoholic beverages have been reported to have medicinal properties of therapeutic value (Bhuyan and Baishya, 2013) with the cultivation of rice being the most common and staple food in many of the regions. Typically, during starter preparations, the mixture is dried under sunshade for 1–2 days to turn it into powder, to be combined in a vessel filled with water. A previously prepared *pitha* is put into the vessel as a yeast source for the cake preparation and is kept wrapped in banana leaves (*Musa paradisiacal* Linn.) for four to 5 days. The dried up cakes (*vekur pitha*) serve as an important source of *Saccharomyces cerevisiae*. Bora rice is also used to make rice beer, which is boiled and kept open on a plate for 1 h before being mixed with *vekur pitha*. The combination is then put inside an earthen pot (referred as a *kalah*). The pot is maintained in a corner for 4–5 days under anaerobic conditions. The alcoholic beverage is then recovered through a filtration method. *Xaj-pani* has a strong scent and gives an alcoholic and sweet taste (Saikia et al., 2007).

Since these techniques and methods are passed from generation to generation, the rice-based beverages do not have any specific or well-defined approach in its manufacture (Das et al., 2012) and is yet to be standardized for commercialization. In this study, we focus on the rice beer preparation done by the Ahom Community of Assam which is deemed as among the favorite. The drink is prepared by rice fermentation with a mix of rare herbs and plays an important role in the socio-cultural lives of the people. Its mild consumption seems to bring relaxation and impart good therapeutic values as it helps in tackling anxiety, depression, stress which uplifts the overall mood of the hardworking population without any reported major side effects.

**FIGURE 1:**
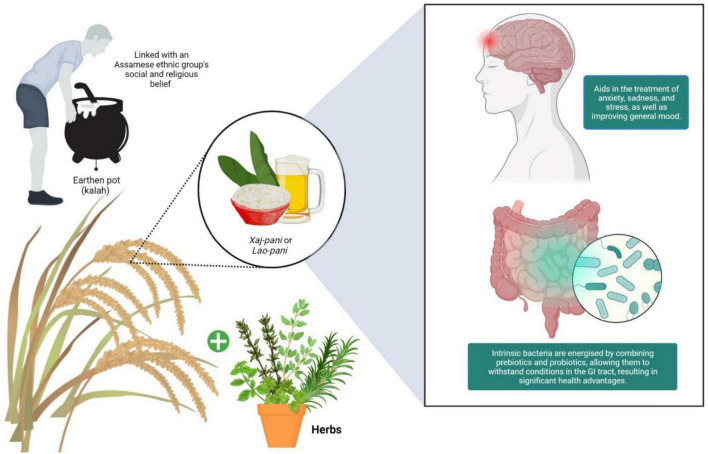
Synbiotics are well-known for their therapeutic effects, which include assisting in the management of mental health issues like anxiety, depression, and stress by elevating general mood.

Synbiotics is a cocktail of prebiotics and probiotics that can potentially influence the gut microbiota; hence the gut microbes regulate mental health and the state of mind (Figure 1). Adoption of probiotics in commercially available fermented food/drinks to prevent various diseases instead of seeking them through the development of probiotic drinks at household scale was unavoidable in a country like India (Lakshmy et al., 2018). In recent times, the most prominent market segment for probiotics has been liquid products. In fact, probiotics in the form of fluid, have been well received by newborns and customers who have problems swallowing pills. To date, novel goods are commercially available, such as yogurt-based beverages and probiotic-infused juices, that provide consumers with new probiotic resources and are believed to provide a significant health benefit (Garcia et al., 2020). Currently, trends are focused on new therapeutic strategies like the development of synbiotics to induce maximum health benefits to the host. Such synbiotics are the combination of prebiotics and probiotics that vitalizes the endogenous microbes and ensure their survival in unfavorable conditions in the gastrointestinal tract (Olveira and González-Molero, 2016). This analysis focuses on potential cereal-based probiotic beverage derived from fermented rice beverages, as well as the health advantages of cereal-based inclusion of probiotic and prebiotic as a synbiotic beverages for human consumption.

## Methods

**FIGURE 2:**
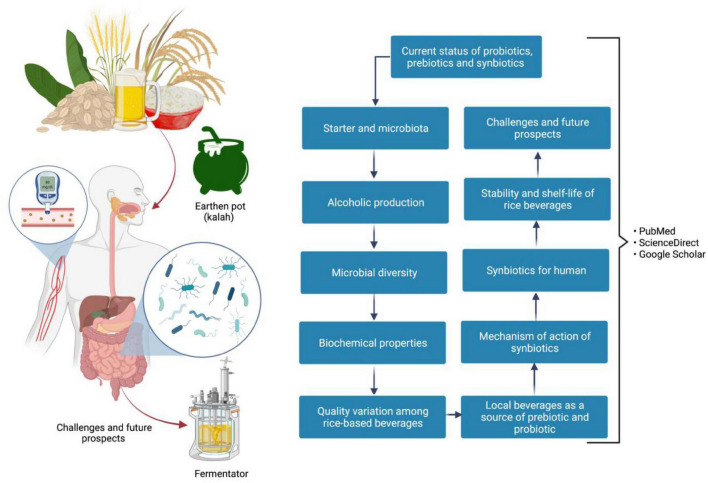
Flow chart of literature review.

Figure 2 depicts the detailed flow of the literature review.

### Current Status

Fermentation is among the earliest biotechnological techniques used for the discovery and synthesis of desirable food products with enhanced sensorial characteristics, palatability and storage life (Ray and Joshi, 2014). The fermented rice beer beverage is unlike any other beverage found in other regions of India because it uses indigenous microbe’s specific to the region. Microbial population consisting of *Saccharomyces, Candida sp.*, lactic acid bacteria (LAB), and *Bacillus sp.* are found to be thriving in the fermentation process of these beverages including Xaj-Pani, Apong, Jou, Judima (Das and Deka, 2012). Cereals are also potentially suitable substrates because (1) they provide nutrients that are easily absorbed by probiotics (Martins et al., 2013; Herrera-Ponce et al., 2014), (2) act as an excellent *Lactobacilli* transporters through the gastrointestinal tract, and (3) accelerate the growth of axenic and co-culture fermentations of probiotic microbes (Charalampopoulos et al., 2003; Charalampopoulos and Pandiella, 2010; Rathore et al., 2012). Lacto fermentation can be carried out either by natural or starter culture, resulting in lactic acid formation. During the lactic acid fermentation process, numerous metabolites including lactic acid, carbon dioxide, ethanol, hydrogen peroxide, acetic acid and antimicrobial peptides (bacteriocin) have been produced from the laboratory, allowing synergistic inhibition and advancement of pathogen-causing microorganisms (Di Cagno et al., 2013).

Cereals can be used to design cereal-based fermented beverages should the formulations satisfy probiotic requirements while also having acceptable physicochemical and sensorial properties (Salmerón et al., 2015). Despite the many challenges in preparing various fermented food, it is apparent that traditional and cultural knowledge of the NE ethnic people when preparing fermented products is preserved among its people, to which it presents extensive opportunities for improving and enhancing product quality (Das et al., 2016). Diversified way of rice beer (like Jou, Apong, Bhaati jaanr, Judima) preparation have the common backbone of fermentation of rice but, the preparation and procedure involved, are unique to each regional community. The traditional starter culture consists of various medicinal plant and their extracts along with microorganisms that initialize and controls the taste, aroma and alcohol content of the beverage. A recent study suggests the good health promoting activities of the microflora of rice beer fermentation, by producing bioactive metabolites that is related to promising therapeutic values (Roy, 2020). Further, detailed microbial load and population dynamics were studied on Xaj-pitha, under controlled fermentation and it designates the population change of microbes on a 7-day fermentation process of Xaj-pani preparation. It is found that there is initial increase in population of fungus, yeast and most importantly LAB along with Enterobacter, with the highest count of LAB recorded on the 4th–5th day of fermentation, along with massive decrease in Enterobacter population after 3rd day. Fungal population is also decreased after the 5th day (Keot et al., 2020).

**FIGURE 3:**
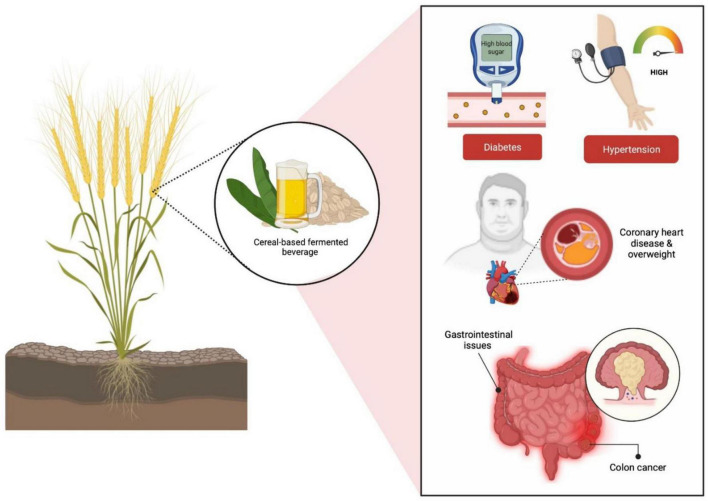
Cereal-based fermented prebiotics have been associated to a reduction in illnesses such as obesity, diabetes, coronary heart disease, and gastrointestinal disorders (colon cancer).

Prebiotics are described as food components comprised of natural fibers that are not metabolized in the upper gastrointestinal (GI) tract and can strengthen host health by preferentially supporting the development as well as the activity of specific genera of microbes in the colon, primarily *Lactobacilli* and *Bifidobacteria* (Gibson and Roberfroid, 1995; Patel et al., 2014; Pandey et al., 2015). In fact, the addition of cereal prebiotics into human diet has been associated with reductions of conditions including diabetes, high blood pressure, coronary heart disease, overweight and gastrointestinal issues such as colon cancer (Berger et al., 2014; Figure 3). A selected blend of probiotics and prebiotics leads to synbiotics which have been shown to boost the viability of triggering the metabolism of health-promoting bacteria, especially *Lactobacilli* and *Bifidobacteria* in the upper and lower GI tracts (Pandey et al., 2015).

Synbiotics alter the microbiota of the gut and promote microbial associations with the immune system and gut epithelium. They may also be useful in treating autism and in studies indicating the gut–brain connection (Vyas and Ranganathan, 2012). Nikbakht et al. (2018) demonstrated through clinical research that probiotic or synbiotic administration may help to avoid or reduce high blood glucose levels in diabetic and non-diabetic individuals since the gut microbiota alteration enhances glucose absorption by generating insulinotropic polypeptides and glucagon-like peptides. Moreover, numerous researchers have revealed that probiotics and synbiotics have no considerable effect on the fasting blood glucose levels (Nikbakht et al., 2018). Consequently, there is a tremendous opportunity to conduct trials with multiple probiotic bacteria and cereal prebiotics to determine if there are viable synbiotic cereal beverages that can dramatically increase blood glucose levels. Additionally, the utilization of probiotic/synbiotic formulations as critical disease therapy necessitates additional research because human trials have confirmed that probiotics have a stronger impact on infections over synbiotics. The use of the 16S rRNA gene sequencing approach can also increase the quality of research confirming the influence of probiotics or synbiotics on rebuilding the microbiome directly by providing more gastrointestinal health benefits following critical disease treatment (Manzanares et al., 2016).

Combination of prebiotics and probiotics have been shown to alleviate psychiatric diseases such as panic disorder, generalized anxiety disorder, severe depression and altered brain function induced by irritable bowel syndrome (IBS) (Pusceddu et al., 2018). Psychobiotics are dietary ingredients that alter the brain and behavior by modulating the gut-brain axis (Sarkar et al., 2016). Furthermore, polyphenols and their constituents in wine can improve the stress-induced neural response and reduce anxiety. Therefore, these metabolites alter the gut microbiota, resulting in healthy host physiology (Zorraquín-Peña et al., 2019). Although diet is a major component in influencing the brain and behavior, incorporating psychobiotic components may be beneficial in the prevention and treatment of some neurological illnesses (Luna and Foster, 2015). Fermented beverages have evolved from traditional natural fermented products to beverages formulated with bioactive components to activate cardiovascular benefits and strengthen gastrointestinal health, which can then be further developed into fermented foods designed with specific bioactivity.

Cereal substrate employed in formulations plays an important role in the production of cereal-based fermented drinks. In fact, rice beverages play an important role in the socio-cultural life of the tribal people as it is considered sacred for offering to God and is used in other religious ceremonies including childbirth, marriages, festivals, and even funerals. Tribal people believe that the utilization of medicinal herbs in preparations of starter culture is important as it possesses medicinal properties which help to ameliorate many health problems (Saikia et al., 2007). To date, a wide variety of fermented food is produced and consumed throughout the state of Assam by various tribal communities, representing a very valuable cultural heritage of each community. Rice-based alcoholic beverages are known by different names such as *Haria* (Adivasi community), *Zu/Judima* (Dimasa tribe), *Arak/Hor-alak* (Karbi tribe), *Jou/Jou-Bishi*, *Jumai* (Bodo tribe), *Apong* (Mishing tribe), *Laopani* (Lalong tribe), *Xaj* (Ahom Community), *Suze* (Deori tribe), and *Choko* (Rabha tribe) (Deori et al., 2007; Das et al., 2012). To date, although many researchers have investigated traditionally prepared rice beverages, limited information is available on the commercialization of beverages like Sake of Japan. At present, traditional fermented rice beverages are prepared for local consumption only (at household level) without much consideration about Good Manufacturing Practices (Mishra et al., 2019). It is generally prepared by adding varieties of medicinal herbs in the starter culture inoculum which is are also rich in microbial population of yeasts, molds and LAB. These microorganisms result in the formation of alcohol which is consumed by the local tribal communities without knowledge on its quality and shelf life. Furthermore, literature has revealed that fermented food products are a good source of amino acids and peptides (Majumdar et al., 2016). Besides, fermentation may also help in the detoxification of certain undesirable compounds like tannins, phytates and polyphenols which are formed or present in our body due to consumption of various raw foods. It is also reported that fermented foods help to control cholesterol, cancer, blood pressure and diabetes as well as boosts the immunity and longevity (Sekar and Kandavel, 2002).

The diversity of raw materials used by different communities mainly contributes to the overall quality and volume of the fermented product. Additionally, the type and variety of rice (glutinous or non-glutinous) used in the preparation greatly influences the aroma, taste and quality of the beverage. Glutinous rice being a rich source of starch protein and various microelements is more preferred as a good source of microbes for causing fermentation (Que et al., 2006). Generally, the microbial population yield hydrolytic and proteolytic enzymes which then assimilate fatty acids, amino acids and discharge simple sugars, ultimately softening food texture. The microorganisms that secrete enzymes like pectinase and cellulase affect food texture to release sugars that is mostly absent for the human digestive system, facilitating easier digestion (Paredes-López et al., 1988). Although the preparations of rice beer by different ethnic communities of NE India is nearly similar; the ingredients remain different, since different plant species have been used, based on the region and availability (Tanti et al., 2010). It is hoped that our study can fill the knowledge gap present.

### Starter and Microbiota

A diverse group of microbes from plant origin as endophytic organisms are the functional microbes for multi-stage fermentation. Apart from these, herbal products are a good source of therapeutic and preservative metabolites that can add extra flavor to the rice-based fermented products (Roy et al., 2004). A prepared starter cake consists of various parts of plants of specific plant species unique to the local community. The cake “vekur-pitha,” translates into a rice cake containing microorganisms. It is prepared by using a unique recipe of dried plant leaves along with rice flour, dried for several days and kept stored for 1 to 2 years.

Previously, a polymerase chain reaction-mediated denaturing gradient gel electrophoresis (PCR-DGGE) has been conducted on traditional Vietnamese alcoholic fermentation starter called *Banh men* indicated the presence of 13 species of fungi that includes yeasts and 23 species of bacteria (Thapa and Tamang, 2004). Additionally, species of amylase-producing Bacillus, acetic acid bacteria and plant pathogens/environment contaminants were also detected. The persistent presence of opportunistic contaminants emphasizes the significance of thoroughly analyzing the effect of individual components within starters. Analysis of starter culture and beverages revealed that *S. cerevisiae* is the principal fungi involved in alcoholic fermentation while for other yeast and mold varieties, LAB have been reported to ferment these beverages. A wide range of microorganisms commonly involving filamentous fungi, enzyme and alcohol generating yeast, LAB, *Bacilli* and *Micrococci* are the chief fermenting microbes (Tamang et al., 2012). *S. cerevisiae* is the principal fungi involved in the fermentation of various yeast and mold. Analysis of starter culture cake collected from Arunachal Pradesh reported the presence of *S. cerevisiae, Hanseniaspora* spp., *Kloeckera* spp., *Pichia* spp., and *Candida* spp. with *S. cerevisiae* deemed as the dominant one (Bhuyan and Baishya, 2013). Besides fungi, yeasts are the major ethanol fermenters especially in case of a cereal-based alcoholic fermentation. Yeast partially breaks down sugars and produce carbon dioxide and ethyl alcohol under anaerobic conditions. Alcohol dehydrogenase which controls the conversions between acetaldehyde and ethanol, is important in ethanol production and assimilation (Lin et al., 2010). On the other hand, amylolytic yeast like *M. circinelloides, R. chinensis, S. capsularis, S. fibuligera*, and *P. burtonii* found in Marcha (fermented product of Sikkim) can degrade starch and produce glucose and then the alcohol producing yeast grow rapidly to produce fermented ethanol.

Lactic acid bacteria strains like *Pediococcus pentosaceus, Lb. plantarum*, and *Lb. brevis* have been isolated from Hamei and Marsha, which are starter culture for the preparation of rice beer in Manipur and Sikkim (Tamang et al., 2007). Another starter called *Apong* (Ipoh) has been reported to contain yeast species like *S. cerevisiae, Hanseniaspora* spp., *Kloeckera* spp., *Pichia* spp., and *Candida* spp. The microbes associated with *murcha*, starter of *kodo ko jaanr* has been identified as *Mucor cicinelloides, Rhizopus chinensis, R. stolonifer var. Lyococcus, S. cerevisiae, S. bayanus, Hansenula anomala, Pediococcus pentosaceus, Lb.* spp., *Candida glabrata, Saccharomycopsis capsularis, S. fibuligera, Pichia burtonii, Pichia anomala*, LAB like *Pediococcus pentosaceus, Lb. bifermentans* (Thapa and Tamang, 2004). Additionally, the presence of filamentous molds like *Mucor circinelloides, Rhizopus chinensis; yeasts Saccharomycopsis fibuligera, Pichia anomala, S. cerevisiae, Candida glabrata*, and LAB- *Pediococcus pentosaceus and Lb. bifermentans* in *Bhaati jaanr* starter have also been reported (Tamang et al., 2007). Moreover, different plant parts like the leaves, bark, fruits of *Holarrhena pubescens, Wattakaka volubilis, Ichnocarpus frutescens*, and *Clerodendrum viscosum* that are used to make *Chullu*, a starter culture made in West Bengal and the fermented product of it, has great ethnomedicinal value among the ethnic group living in the Malda District (Saha et al., 2015). Furthermore, *Pediococcus pentosaceus*, *Enterococcus faecium*, *Lb. curvatus*, *Weissella confuse*, and *W. paramesenteroides* were predominant LAB of a dry-starter, ragi tape found in a Balinese rice beer (Sujaya et al., 2001). Studied probiotics such as *Escherichia coli* and LAB have been evaluated to prevent a variety of intestinal disorders (Behnsen et al., 2013). The presence of some LAB strains along with the potential beneficiary substances (mannobiose, sugar alcohol, amino acids) produced by the microbial consortia during rice beer fermentation may provide health benefits (Das et al., 2019b).

### Alcohol Production

At the beginning of the fermentation process, amylolytic fungi degrades starch to dextrin in the presence of α-amylase which is further hydrolyzed to glucose by glucoamylase. Then, alcohol-producing yeast (mainly *S. cerevisiae*) take over the fermentation process which also supplements the product with a variety of vitamins, amino acids and helps in creating the flavor and aroma (Ghosh et al., 2015). Ethanol produced by *S. cerevisiae* also affects LAB population and reduces it during the first stage of fermentation, although LAB can tolerate the increasing presence of ethanol that is released during storage. Yeast cell lysis continues in the presence of nutrient availability allowing, LAB to grow (Lonvaud-Funel, 1999). Apart from it, *Meyerozyma guilliermondii, Wickerhamomyces ciferrii, Candida glabrata, Debaryomyces hansenii, Ogataea parapolymorpha*, and *Dekkera bruxellensis* are also reported to be ethanol producers. Additionally, microbial population also consists of amylase producers like *Rhizopus delemar, Mucor circinelloides*, and *Aspergillus* spp. in *Xaj* starter cake. Again, the bacterial population is chiefly dominated by LAB. Some yeasts are responsible not only for ethanol and gas production, but also for the flavor and sensory qualities like taste and aroma as they produce certain group of complexes like glycosides, fuel alcohols, acids and esters (Aidoo et al., 2006).

### Microbial Diversity of Xaj-Pani

Fermented food includes and contain microbes from the surroundings consisting of mycelial or filamentous molds, yeasts and bacteria. Microorganisms, which are found in/on the ingredients, plant and animal sources, containers and the environment are selected through adaption to the substrates (Hesseltine, 1983; Steinkraus, 1996). Generally, three major groups of microorganisms are found in the fermented beverages: bacteria, yeasts and fungi. Normally, traditionally prepared cereal-based fermented alcoholic beverages is carried out in two major steps (1) enzymatic breakdown and (2) hydrolysis by fungi from starch to glucose. The subsequent process is followed by fermentation by yeast (fewer fungi) from sugar (glucose) to ethanol (Nout and Aidoo, 2011).

A previous study describes the microbial diversity of rice beer, where the next-generation sequencing analysis of 16S rRNA amplicons was carried out and analyzed with the QIIME pipeline (Caporaso et al., 2010). Alpha diversity that measures species-richness and evenness within a sample was determined considering the observed operational taxonomic units (OTU), Faith Phylogenetic Diversity (FPD), Shannon’s diversity index and Pielou’s Index (PE). In the study, three samples of *Xaj* (beer) were collected from three different regions in Assam. The observed OTU, FPD, Shannon’s Diversity index and PE indicate the unique number of features, community richness, incorporating phylogenetic relationship between features, community richness and community evenness, respectively. In the study, *Leuconostoc* was the dominant microorganisms and others like LAB, *Acetobacter, Acinetobacter, Bacillus, Dickeya, Enterococcus, Enterobacter, Exiguobacterium, Gluconobacter, Janibacteria, Rothia, Klebsiella, Pseudomonas*, and *Staphylococcus* were also present (Das et al., 2019a).

Three amylolytic filamentous fungi namely *Rhizopus delemar, Mucor circinelloides* (Family*: Mucoraceae*), and *Aspergillus sp*. (Family: *Trichocomaceae*) co-exist in the starter cake. Again, yeasts microbial community play an important role in alcoholic fermentation. The major yeast species found in *Xaj* starter cakes are *Meyerozyma guilliermondii, Wickerhamomyces ciferrii, S. cerevisiae, Candida glabrata, Debaryomyces hansenii, Ogataea parapolymorpha*, and *Dekkera bruxellensis*. Furthermore, various other plant pathogens were reported to be present in the starter cake like *Acidovorax avenae, Acidovorax avenae subsp. avenae (Pseudomonas avenae), Acidovorax avenae subsp. citrulli, Herbaspirillum seropedicae, Herbaspirillum* sp. *GW103, Pantoea, Methylobacterium, Sphingomonas*, and *Xanthomonas*. Additionally, environmental contaminants like *Pseudomonas fluorescens, Pseudomonas* sp. *CBZ-4, Pseudomonas stutzeri, Pseudomonas aeruginosa group*, and *Stenotrophomonas maltophilia* were also detected (Bora et al., 2016). Several opportunistic human skin commensals, such as *Acinetobacter guillouiae, Microbacterium* sp., *Micrococcus* sp., and *Staphylococcus* sp. were examined but among these, *A. guillouiae* has received increasing attention, as significant opportunistic pathogen, usually in the context of causing serious underlying disease in immunocompromised patients from South-East Asia and tropical Australia (Dijkshoorn et al., 2007; Peleg et al., 2008). The different types of contamination were assumed to be introduced in the addition of plant parts or through unhygienic practices.

### Biochemical Properties of Xaj-Pani

According to a fermentation study on Vietnamese rice-based alcoholic beverages, glucose concentration increases on the first 3 days of the fermentation process but is used up mostly toward the end of the fermentation process where ethanol content start to rise gradually (Dung et al., 2005). During the fermentation process, the formation of organic acids grants the flavor and increases the shelf life by decreasing the pH of the rice beer which interferes with growth of LAB (Vandenbergh, 1993).

Based on an analytical study on three samples of rice beer (Das et al., 2019b), they were reported to have high antioxidant activity. Alcohol content was estimated by following potassium dichromate method with some alterations and ranged from 9.41 to 14.82% (Caputi et al., 1968). Phenolic content of the samples was measured using Folin-Ciocalteu (FC) method and ranged from 2.64 to 4.14 mg/ml (Singleton et al., 1999). The antioxidant activities of the rice beer were investigated following 2,2-dipheny 1-1-picrylhydrazyl (DPPH) method (Ghosh et al., 2015). Determination of antioxidant activity was accompanied by 2,2′-azino-bis (3- ethylbenzothiazoline-6-sulfonic acid) and ABTS assays with some slight modifications (Ghosh et al., 2015). Phenolic content of the samples was determined using the FC method (Singleton et al., 1999) and was found to be ranging between 2.64 and 4.14 mg/ml. One of the Xaj samples showed a high antioxidant activity (4.14 mg/ml) while another sample showed low antioxidant activity (1.69 mg/ml). Similar data was observed in case of free radical scavenging activity against ABTS. The antioxidant activity varied from 1.69 to 3.91 mg/ml of ascorbic acid. Overall, the radical scavenging effects and phenolic content analysis of *Xaj* rice beer starter cake is seen to be higher as compared to some other locally prepared rice beverage starter cakes like *Apong* and *Sujen* (Handique, 2019). Hence, *Xaj* rice beverage consumption is good due to its high antioxidant activity.

### Quality Variation Among Rice-Based Beverages

The diversity and distinction of rice beer beverages from Assam has been reported in details (Das et al., 2012; Bhuyan et al., 2014). For example, the alcoholic content of rice beers including *Apong, Xaj, Joubishi* have been investigated and was found to vary between 9.41 and 19.33%. Similarly, *Apong* contained highest microbial diversity and species richness whereas *Xaj* has the least bacterial diversity which may again be advantageous to the host. Furthermore, the culture-independent study leads to the existence of 18 core bacteria in which LAB involving *Lb., Leuconostoc, Pediococcus, Lactococcus*, and *Weissella* had higher abundances in all three types of rice beers (*Apong, Xaj*, and *Joubishi*) (Das et al., 2019b). The traditional method of beverage preparation varies within community due to the lack of well-defined recipe leading to the eventual diversity in aroma, taste, and alcoholic percentage. The tribal people of Tripura also use different plant varieties in making the rice beer; however, the availability of plants, rice varieties and variation in plant species confers taste and flavor variation (Ghosh et al., 2015). Additionally, the type and quality of rice used for the fermentation process also greatly influences the quality of the rice beer. For example, glutinous rice contains a rich basis of protein, starch and various microelements that allows microbes to ferment more alcohol (Que et al., 2006). Nevertheless, due to the traditional techniques that lack defined methodology and recipe, quality variation is observed even within the same type of rice beer from the same community. In a study, probiotic bacteria such as *Pediococcus pentosaceus* and *Pediococcus anomala* strains were isolated from *Hamei*, an ethnic rice beer (called *Atingba*) fermentation culture of Manipur, India. The use of these microbes in the starter showed positive effect on the beer quality with an overall improvement in terms of their microbiological properties as well as biochemical properties (Mangang et al., 2017b). During the 3 months that the beers were stored, there was a greater inhibition of lipid peroxidation. As a result, the beers differ depending on the type of microbial starter employed, with wild-type cultures being preferred (Mangang et al., 2017b).

## Rice Beverage as a Source of Pro- and Prebiotics

### Perspective of Pro-, Pre- and Synbiotic Beverages

Probiotics are defined as live microbes provided in adequate amounts to confer health advantages to the host (FAO/WHO, 2002). Probiotics are now regarded as one of the nutraceutical treatment strategies for the control and prevention of a variety of chronic disorders related to digestive and immunological health. Furthermore, probiotics have been regarded as live microbial feed supplements which improve the GI microbial balance of the host and also provide health advantages other than offering basic nutritional benefit (Nagpal et al., 2012; Adnan et al., 2017; Alshammari et al., 2019). Interestingly, probiotic supplementation aids in the restoration of thymus histology and activates the adaptive immune response (Núnez et al., 2013). Probiotic bacteria and bioactive substances found in fermented foods have antiviral properties against viruses of the respiratory and gastrointestinal systems. The mechanism of action was claimed to be attributable to the stimulation of immune system activity by raising natural killer cell toxicity, increasing pro-inflammatory cytokines production, and increasing T lymphocyte cytotoxicity (Muhialdin et al., 2021). Probiotic is also known to accelerate vaccine response (Zimmermann and Curtis, 2018) where their immunomodulatory roles may be advantageous in COVID-19 infection. In fact, it has been reported that the potential of probiotics to control gut microbiota may in turn, regulate the immune system in a way that could be beneficial in COVID-19 (Adnan and Pramaningtyas, 2020). The local residents have also traditionally made such beverages, which have kept a unique microbiota over the years. Ethnic fermented beverages are being explored in order to discover new probiotic bacteria and biologically effective compounds (Mishra et al., 2018; Sahu and Panda, 2018; Ghosh et al., 2019). Fermented drinks derived from cereals and legumes can aid in the production of a better functional beverage (Das et al., 2016; Panda et al., 2018). Another study was conducted to isolate and characterize the effects of traditionally prepared probiotic-containing functional fermented rice beer of Assam, where it was found that the isolates were gamma-hemolytic in nature and the cell-free supernatant concentrate displayed no toxicity against mouse liver cells (Borah et al., 2019). In another study, Ghosh et al. (2015) used the probiotic strain *Lb. fermentum* KKL1 to make the rice-based fermented drink that released enzymes (amylase and phytase) and also show some antioxidant properties. Some studies have also found that traditional African fermented cereal-based drinks are putative probiotic carriers owing to the presence of probiotic *Lb.* spp. and yeasts involved in their fermentation.

The World Gastroenterology Organization highlighted that the effectiveness of probiotics is strain- and dose-specific (McFarland, 2015). Whole grain cereals including cereal components offer a probiotic carrier alternative while also ensuring efficient bioactive components and fiber (Waters et al., 2015; Enujiugha and Badejo, 2017). Such components involve non-digestible carbohydrates, soluble fiber and phytochemicals such as phytoestrogens, antioxidants, phenolic compounds and phytic acids (Waters et al., 2015). Despite being the world’s primary source of dietary nutrition, cereal grains lack certain essential food elements such as amino acids (Marsh et al., 2014). Fermentation can improve the nutritional contents, sensory properties and functional aspects of cereals (Navarrete-Bolaños, 2012).

**FIGURE 4:**
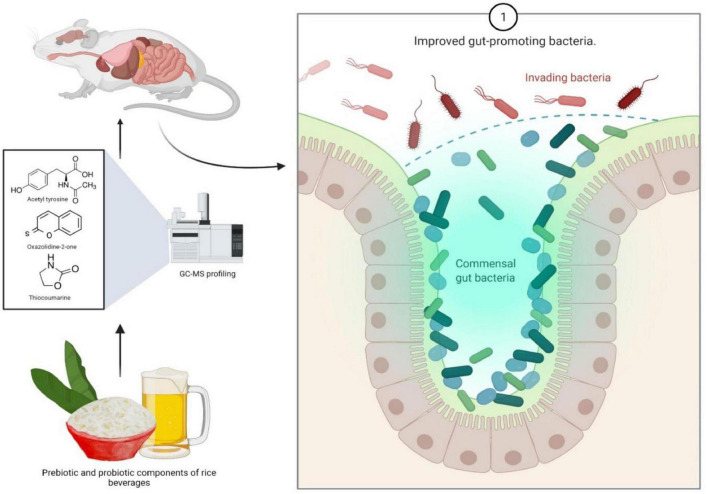
Rice beer appears to contain bioactive molecules, indicating the presence of prebiotics, which have a number of health benefits, including the ability to fight infections and regulate gut flora.

The human gut system comprises an enormous indigenous population of bacteria with more than 1,000 species of bacteria that maintain an important role in immune homeostasis, colonization resistance against pathogens and also maintaining absorption of food by the host (Guarner and Malagelada, 2003). Probiotics are studied as an alternative therapy against drug-resistant pathogens (Forestier et al., 2001). They also have properties like maintaining good digestive health, reduce gut-related disorders and heart diseases (Borruel et al., 2002). Probiotic bacteria like *Lb., Leuconostoc, Pediococcus, Bifidobacterium* show adversary effects on pathogenic genera like *Clostridium, Salmonella, Shigella, Escherichia, Helicobacter, Campylobacter, Candida*, etc., (Collado et al., 2007; Figure 4). In fact, the diverse types of rice beer prepared by the ethnic community of NE India have probiotic potential which remains under-explored (Saikia et al., 2018). The Indigenous fermented rice beverage is rich in microbial diversity which could be promising probiotics.

Studies have revealed that fermented food products are rich in microbial diversity. Mucor circinelloides, Rhizopus chinensis, Saccharomycopsis fibuligera, Pichia anomala, Saccharomyces cerevisiae, Candida glabrata, Pediococcus pentosaceus, Lb. bifermentans were isolated from rice beverage, Bhaati jaanr from Sikkim (Handique and Deka, 2016). LAB and yeast could be used further as probiotics which will impart health benefits to the consumer. Bernal-Castro et al. (2019) examined the efficacy of commercial probiotics (Lb. casei) in the production of a functional tropical/red fruit drink containing 20% strawberry, 10% blackberry, and 5% papaya with the addition of prebiotics (inulin and fructooligosaccharides). The growth kinetics (37°C for 50 h) were used to measure the vitality of Lb. casei added into the fruit beverage. The analysis indicates that fortifying the fruit beverage with 1% inulin can protect the stability of Lb. casei. Some analyses are also conducted to assess the impact of probiotics in controlling COVID-19 by improving the host immunity and decreasing pathogen association in the host. Further research suggests that probiotics may act against virus along with other infections (Olaimat et al., 2020; Tiwari et al., 2020). In fact, the use of probiotics, their metabolites and prebiotics were regarded to be a viable therapeutic strategy for a variety of infectious and non-infectious ailments (Markowiak and Śliżewska, 2017; Silva et al., 2020).

As opposed to probiotics, prebiotic is defined as a non-digestible food component that provides nutritional support to noble microbes existing inside the gut, to stimulate their growth and ameliorate health for both human and animals (Gibson et al., 2004; Bindels et al., 2015). It is confirmed that gut microbiota has positive effects on host’s health. Therefore, recently prebiotics are used to manipulate gut health. Prebiotics (non-digestible fiber components) present in fruit and vegetable beverages continue to support and stimulate the growth of probiotics in the gastrointestinal tract. Pimentel et al. (2015) investigated the effect of oligofructose fortification on the physicochemical parameters of apple juice and the survivability of the probiotic (*Lb. paracasei*) during storage at 4°C for 28 days. Interestingly, the use of oligofructose as a prebiotic elevated the survivability of the probiotic (*Lb. paracasei*) in apple juice. Prebiotics are defined by FAO/WHO scientists in 2007 as non-viable food ingredient which delivers benefit to the host *via* microbiota regulation (FAO/WHO, 2002). Additional substances with prebiotics such as fructo-oligosaccharides, galacto-oligosaccharides and isomalto-oligosaccharides may enhance the growth of beneficial bacterial in the gut and hence can be used as a treatment of some diseases such as diabetes, non-alcoholic fatty liver disease, antibiotic-associated diarrhea, colitis, constipation, cancer, hepatic encephalopathy, food-borne illness, hypercholesterolemia and colorectal cancer.

Other than the gut, probiotics and prebiotics have also been orally introduced for skin health (Foolad and Armstrong, 2014). For example, the presence of potent prebiotics like cellobiose and mannobiose in metabolite also enhances the nutritive value (Das et al., 2019b). In fact, the combined use of probiotics and prebiotics can improve human or animal health. Gibson and Roberfroid coined the word “synbiotic” in 1995 to describe a mixture of probiotics and prebiotics which act synergistically (Gibson and Roberfroid, 1995). A specific component introduced into the GI tract must be able to specifically encourage the growth and/or activate the physiological microbiota of the intestine, thereby boosting the host’s health (Skalkam et al., 2016). Synbiotics provide both probiotic and prebiotic characteristics and are developed to circumvent some of the potential challenges for probiotic survival in the GI tract (Rioux et al., 2005). As a result, combining both components into a single product lead to a higher benefit when compared to the individual efficacy of pro- or prebiotic (Panesar et al., 2009).

Synbiotics are considered to be an important factor that influences emotional disorders and the immune structure. Furthermore, they also confer some effects in the regulation of the neuroimmune regulation and control axes in the nervous system diseases. Following ingestion, probiotic bacteria is concentrated in the intestinal epithelium, following which they may also produce neuroactive elements that act on the brain-gut axis (Bermúdez-Humarán et al., 2019). To date, it has been confirmed that there is a bi-directional link or connection between the intestine and the brain involving neurological, metabolic, hormonal and immunological signaling pathways where its alteration can bring altered behavior in these systems (Rhee et al., 2009). Not only it serves as a source of probiotic elements, but it is confirmed that ethnic beverage contain more proteins, carbohydrates and nutritive value when considered against foreign alcohol (Arjun, 2015). A recent study reveals how probiotics and prebiotics content in rice beer shows effects on human gut microbiota and fecal metabolites (Deb et al., 2020).

In another study, the effect of rice beverages on gut microbiota and health has been determined by using a mouse model (Bhaskar et al., 2021). The study indicates sundry effects of pre- and probiotic components of rice beverages, hence the treated mice were found to have healthy gut promoting bacteria. Another similar study reveals the positive effect of components of rice beverage on anxiety, behavior and spatial memory in a mouse model (Bhaskar et al., 2021). The metabolite content of Rice beer varieties of Assam mainly consisted of 18 saccharides, 18 organic acids, 11 sugar alcohols, eight amino acids, a vitamin and several nutraceutical compounds including thiocoumarine, oxazolidine-2-one and acetyl tyrosine done by gas chromatography mass spectroscopy-based profiling, suggesting the presence of potent probiotics and nutraceuticals in rice beer, hence confirming these health benefits (Das et al., 2019b). Probiotics are also used as a treatment or preventive measure against some gastroenterological conditions like IBS, infectious diarrhea including noso-comical infections, inflammatory bowel disease (IBD), necrotising enterocolitis (NEC), as well as cancer and to protect the adverse effects of chemotherapeutic agents. Its impact is also an indicator of health as it influences the incidence of common infectious disease, risk of allergy, improvement in bowel function and modulation of immune function (Sanders et al., 2013).

Saccharification of steamed rice is involved in the production of rice wine, accomplished by fungal enzymes under aerobic solid-state fermentation. Molds are submerged in water for alcoholic fermentation by several yeast cultures (*Pichia anomala, S. cerevisiae*, and *Trichosporon*) which are allowed to occur with conventional starter flat cakes (Jeyaram et al., 2008). The starter culture employed in rice wine is locally known as *xaj-pitha*, which is a mixture of molds, yeasts and bacteria (LAB) grown on substrates likes rice powder and supplemented with different herbs. Some of the most commonly encountered LAB strains are *Lb. plantarum*, *Lb. brevis, Leuconostoc lactis*, *Weissella cibaria, Lactococcus lactis, Weissella paramesenteroides, Leuconostoc pseudomesenteroides*, and *Pediococcus pentosaceus* (Bora et al., 2016). LAB is involved when a wide variety of carbohydrates is consumed and in the metabolism of various phenolic compounds. The role has evolved further into providing an appropriate choice for the development of novel healthy plant-based beverages (Filannino et al., 2014; Hur et al., 2014).

The making of starter cake is unique and comprises many valuable medicinal plant parts. The starter cake used in “*Xaj-pani*” is called “vekur-pitha,” prepared using specific plants such as *Centella asiatica, Cinnamomum bejolghota, Cissampelos pareira, Clerodendrum viscosum, Croton caudatus, Hydrocotyle sibthorpioides, Lygodium flexuosum, Naravelia zeylanica, Oryza sativa, Pteridium aquilinum, Piper nigrum, Sida rhombifolia*, and *Smilax perfoliate*. The medicinal herbs added during the preparation of rice beverage contribute to the color and flavor of the final product.

The microbiota also contribute to the flavor and texture of the drink. Aside from adding color, flavor and sweetness to the drink, the plants employed in the starter culture are often believed to harbor a variety of therapeutic benefits. Overall, the quality of the starter culture is affected by the plant parts chosen. With increased emphasis on health as well as the number of vegetarians globally, the demand for plant-based probiotic products has greatly expanded especially in their utility of chronic dysentery, liver disorder, gastric troubles, diarrhea and urinary troubles (Senapati and Gurumayum, 2016) treatments.

**FIGURE 5:**
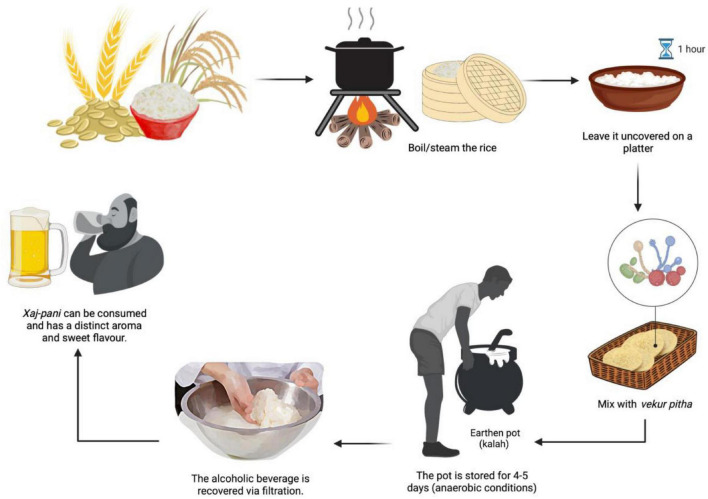
Process of “Xaj-pani” preparation.

Overall, the process of “Xaj-pani” preparation is depicted in Figure 5.

1. Glutinous or non-glutinous rice is half-cooked, before being allowed to cool down on banana leaves which can provide some flavor and aroma to the product.

2. Subsequently, the starter cake “vekur-pitha” is grounded and mixed with the cooled rice. After a uniform mixing is achieved, the mixture is kept for several minutes [the starter culture (weighing approx 30–50 g) in 1 kg of rice].

3. The mixture is kept in an earthen pot with its mouth sealed and is kept in a closed room for 3–5 days.

4. The content is then checked regularly to ensure that it is moist (Water will be added if necessary).

5. After the fermentation period, an adequate amount of water is added and the mixture is allowed to rest. Then, it is filtered through a fine clean cloth using a series of vessels before the extract can be consumed as rice beer (Das et al., 2012).

### Mechanism of Action of Synbiotics

Probiotic and prebiotic foods are functional foods that appeal to humans. The synbiotic effect occurs when probiotics and prebiotics work together. Rice bran is a byproduct of rice milling that is high in nutrients and high in dietary fiber. Dietary fibers such as hemicellulose, arabinogalactan, arabinoxylan, xyloglycan, proteoglycan, arabinofuranoside, and raffinose are present in rice bran. Antioxidants such as oryzanol, tocopherol, tocotrienol, and ferulic acid can also be found in rice bran. This indicates that this product has the potential to be used as a fermentation medium for the production of innovative probiotic beverages.

Probiotic seems to be mostly active in both small and large intestines, while the effect of prebiotic is more marked in the large intestine; the combination of the two may have a synergistic effect (Hamasalim, 2016). Prebiotics are primarily used as a selective medium for probiotic strain proliferation, fermentation and intestinal passage. There is evidence that in the presence of prebiotics, probiotic can help microbes achieve significant tolerance to the environmental factors such as oxygenation, pH and temperature in the intestine (Sekhon and Jairath, 2010). Nevertheless, a higher tolerance to these factors is not sufficiently explained to date. An interaction of these components leads to the synthesis of viable microbial dietary supplements and ensures the maintenance of the host health.

Synbiotics are known to have two modes of action (Manigandan et al., 2012) by (1) improving the viability of probiotic microbes and (2) the provision of exerting specific health effects. Stimulation of probiotics occurs along with prebiotics as it controls the metabolic activity inside the intestine resulting in maintenance of the intestinal biostructure, activating beneficial microbiota and also helping to inhibit potent pathogens residing in the GI tract (De Vrese and Schrezenmeir, 2008). Additionally, synbiotics reduce the quantities of unwanted metabolites by inactivating nitrosamines and cancer-causing agents. Its use has been reported to significantly increase the level of short-chain fatty acids, ketones, carbon disulfides and methyl acetates that may have a positive influence on the host’s health (Manigandan et al., 2012). In terms of therapeutic efficacy, the desired characteristics of synbiotics include antibacterial, anticancerogenic and anti-allergic properties. They also relieve constipation and diarrhea by counteracting the decay processes in the gut. Additionally, synbiotics are efficient in preventing osteoporosis, reducing the level of fat and sugar in the blood, activating immunological system and also treats brain disorders associated with abnormal functioning of the liver (Pandey et al., 2015). It is known that the concept of mechanisms of action of symbiotic is based on the change of intestinal microbiota along with probiotic microbes and also selected prebiotics as their substrates.

### Synbiotics for Humans

**FIGURE 6:**
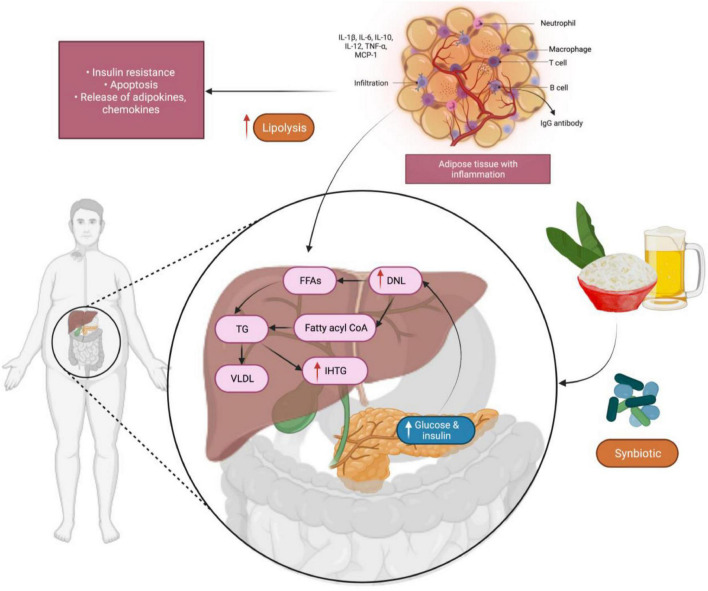
The development of intrahepatic triacylglycerol (IHTG) in the liver, facilitated by a mechanism that enhances the production and storage of liver triacylglycerols. Synbiotics, which are made up of many beneficial probiotics, showed a significant reduction in IHTG level. IL-1β, -6,-10,-12, Interleukin-1 beta—6,-10,-12; TNF-α, Tumor necrosis factor alpha; MCP-1, Monocyte chemoattractant protein-1; FFAs, Free fatty acids; DNL, *de novo* lipogenesis; TG, triglycerides; VLDL, Very low-density lipoprotein.

Synbiotics provide numerous beneficial effects to humans (Zhang et al., 2010) including increasing the levels of *Lb.* and *Bifidobacterium* genus and maintaining the balance of microbiota within the intestine. These are achieved by (1) improving the hepatic function in patients having cirrhosis (2) improving the immunomodulatory activities (3) inhibiting the translocation of bacteria and (4) decreasing the incidence of nosocomial infections in patients undergoing post-surgical processes. Bacterial translocation metabolism products includes ethanol, lipopolysaccharides (LPSs) and short-chain fatty acids (SFCAs), results in their penetration into the liver. SCFAs can enhance the synthesis and storage of liver triacylglycerols that promotes the mechanisms of intrahepatic triacylglycerol (IHTG) and also intensify liver steatosis. A randomized trial was conducted to investigate the use of a synbiotic comprising of five probiotics (*Lb. plantarum, Lb. delbrueckii* spp. *bulgaricus*, *Lb. acidophilus, Lb. rhamnosus, Bifidobacterium bifidum*) and inulin as a prebiotic in adult subjects suffering from liver inflammation and damage due to fat deposition demonstrated a remarkable reduction of IHTG associated with insulin-resistant glucose metabolism within 6 months (Wong et al., 2013; Figure 6). Additionally, LPSs also triggers pro-inflammatory cytokines including tumor necrosis factor alpha (TNF-α) which plays a crucial role in insulin resistance and inflammatory cell uptake in patients with non-alcoholic fatty liver disease. In one study, 52 adults were enrolled for 28 weeks to examine the effect of the synbiotic product consisting of a blend of probiotics (*Lb. casei, Lb. rhamnosus, Streptococcus thermophilus, Bifidobacterium breve, Lb. acidophilus, Bifidobacterium longum, Lb. bulgaricus*) and fructo-oligosaccharides. The researchers established inhibition of nuclear factor κB (NF-κB) and reduced level of TNF-α among the participants (Eslamparast et al., 2014). Additionally, research on animal rat model show increased intestinal IgA, following administration of a synbiotic product containing *Lb. rhamnosus* inulin and *Bifidobacterium lactis* as well as oligofructose as prebiotics when added into the diet. Overall, synbiotics are helpful in lowering blood cholesterol levels and blood pressure (Socha et al., 2002).

Synbiotics can treat hepatic disorders (Pathmakanthan et al., 2002) and also enhance calcium, magnesium and phosphorus absorption (Pérez-Conesa et al., 2006). Dang et al. (2013) carried out a meta-analysis of published research on probiotics and prebiotics for eczema prophylaxis, bacterial strain efficacy and alterations in the allergy status of the children participated. Based on the meta-analysis, probiotics or synbiotics minimize the risk of eczema in infants less than 2 years. Its administration has no effect on systemic sensitization.

The anti-carcinogenic potential of synbiotics were confirmed in studies conducted within the context of the Synbiotics and Cancer prevention in Humans (SYNCAN) project, which has been supported by the European Union. It is a human dietary intervention study to reduce colon cancer risk. SYNCAN project was involved in the integration of an *in vitro* analysis to select a suitable synbiotic formulation and to apply it in an *in vivo* rat model with chemically induced colon cancer. Additionally, the role of prebiotic fructo-oligosaccharides (SYN1) in addition with two probiotic bacteria (*Lb. rhamnosus* GG and *Bifidobacterium animalis* subsp. *lactis* Bb12) in patients having colorectal cancer was investigated. A change in biomarkers like genotoxicity, labeling index, labeled cells/crypt, transepithelial resistance, necrosis, interleukin-2 (IL-2), IFN-γ prevents disease progression in cancer patients and in patients following polypectomy. Furthermore, synbiotic consumption can prevent an increased secretion of IL-2 by PBMCs in the polyp group without showing any effect in the cancer group. In contrast, synbiotic enhances the production of IFN-γ in the cancer group but not in the polyp group (Rafter et al., 2007) indicating its efficacy in the treatment of cancer. Rafter et al. (2007) also investigated the increased number of *Bifidobacterium* in both groups and *Lb*. in patients with polyp, whereas *Bacteroides* and *Enterococcus* were unaffected in both groups. Nevertheless, the number of *Clostridium perfringens* was significantly decreased in patients with polyp. Overall, it was concluded that the application of synbiotics plays a role in reducing the risk of colorectal carcinoma.

Van Loo et al. (2005) in his SYNCAN study reported a lower level of DNA damage and colonocyte proliferation ratio. They demonstrated the functional effect of synbiotic formulation in which probiotic survives during GI transit and modulate the intestinal flora. Additionally, LAB strains and some prebiotics can also prevent carcinogen-induced damage to DNA in the rat colon (Pool-Zobel et al., 1996). Both probiotics and prebiotics have also been revealed to reduce preneoplastic lesions and tumors in the rat colon exposed to chemical carcinogens (Goldin et al., 1996; Reddy et al., 1997). Probiotics can suppress aberrant crypt foci (ACF) (Rowland et al., 1998) and DNA damage in the rat colon (Wollowski et al., 1999). Most of these activities are attributed to its role in scavenging of carcinogenic intermediates, which resulted in less carcinogenic exposure of colonocytes; the antigenotoxic effect could reduce the likelihood of cancer development and progression (Haskard et al., 2001). Furthermore, the chemoprotective impacts of prebiotics have been linked to increased yield of butyrate that hinders colon cancer cell proliferation, acts as a survival factor in healthy colon cells, increases the expression of phase II detoxifying enzymes in both normal and transformed cells and also protects against genotoxic compounds (Abrahamse et al., 1999; Pool-Zobel et al., 2005).

### Immunological Response Involving Symbiotic and Prebiotics

**FIGURE 7:**
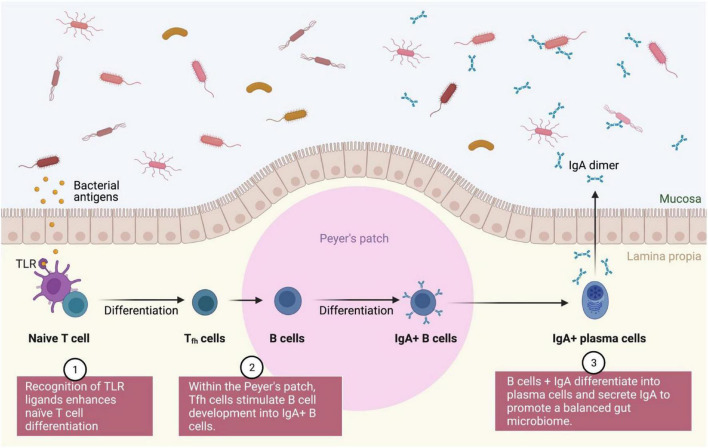
IgA modulated gut microbiota regulation. The interaction between TLR and naïve T cell leads to sequence of differentiation process into plasma cells for modulation of a healthy gut microbiota. TLR, Toll-like receptors; Tfh, T follicular helper; IgA, Immunoglobulin A.

Different immunological pathways (mainly innate and adaptive) continue to be identified for symbiotic and prebiotics. The interactions between innate and adaptive immune cells maintain a balance in the gut microbiota between immunological tolerance and inflammation (Zheng et al., 2020). When a probiotic interacts directly with the intestinal epithelial cells or are totally internalized by M-cells, they cause a variety of immune responses that result in the regulation of pathogen-induced inflammation *via* signaling pathways regulated by the Toll-like receptors (TLR). Intestinal dendritic cells are the primary cells that function as ligands for this receptor (DCs) (Frei et al., 2015). In general, TLR ligand recognition stimulates the development of naive T cells into T follicular helper (Tfh) cells, which in turn promotes the differentiation of B cells into IgA + B cells (plasma cells) within the Peyer’s patch, where immunoglobulin A (IgA) is responsible for regulation of gut microbiota (Lycke and Bemark, 2017; Figure 7). In humans, DCs, stromal cells, macrophages and epithelial cells synthesize *all-trans*-retinoic acid (atRA) from the retinol (Vicente-Suarez et al., 2015), where atRA binds differently with the retinoic X receptor alpha (RXR) and the retinoic acid receptor (RAR) expressed by the three unique genes (α, β, and γ) (Stevison et al., 2015; Qin et al., 2018). Additionally, CD103 + dendritic cells in the lamina propria of the small intestine can facilitate retinoic acid metabolism in response to probiotic bacteria (Konieczna et al., 2012, 2013). The primary source, intestinal mononuclear phagocytes expressing this biomarker and/or CX3CR1, has been shown to play a substantial role in directing immune responses toward gut commensals, inducing inflammation and activating regulatory T cell responses. T helper (Th) cells are activated by I–peptide complexes (Couture et al., 2019), as presented by antigen-presenting proteins such as HLA-DQ and HLA-DR, which are triggered by certain microbiomes and LPS resulting in an increase in the CD80, CD86, and CD40 surface markers. However, the presence of microbiota signals CX3CR1 + inflammatory cells and CD103 + CD11b + DCs to stimulate IL-22 and IL-23 production resulting in successful colitis recovery (Longman et al., 2014). Nonetheless, pro-inflammatory lamina propria-derived TNF-α may worsen colitis *via* the same signals, implying that the DC subset also contributes to the maintenance of balanced inflammatory and/or standby conditions in the gut (Mahapatro et al., 2021).

In another intriguing finding, *Lactobacillus rhamnosus GG*-derived soluble protein, p40, was shown to reduce keratinocyte chemo-attractant, TNF, IFN, and IL-6 production in oxazolone-treated mice, implying that p40 regulates innate immunity, since certain expressions such as IL-1β, IL-10, IL-13, and IL-17 remained unaffected (Yan and Polk, 2011; Javanshir et al., 2021). Numerous probiotic microorganisms, including *Bifidobacterium bifidum* PRL2010 which are frequently found in mammals’ gastrointestinal tracts have been shown to suppress epithelial cell pro-inflammatory chemokine responses (O’Neill et al., 2017). Other mucosal metabolites which can induce IL-10 while reducing pro-inflammatory cytokines include histamines and butyrate which also regulates Th17 responses and promote IL-23 secretion (Frei et al., 2015).

## Stability and Shelf-Life of Rice Beverages

During the 3 months storage period, more inhibition of lipid peroxidation was observed and the difference in the shelf-life was seen for the beers depending upon the type of microbial starter used. Fermented rice beer of Manipur, India known as *Atingba* is prepared from *Albizia myriophylla* bark revealed extended shelf life (32°C for 3 months) for the wild-type culture rather than the established type of microbial starter (Mangang et al., 2017b). Moreover, researchers found utilized them in the food industry as a natural food supplement and a preserving agent. Another study suggests that flavonoid extracts improve the shelf-life stability of rice beer under accelerated conditions (Das et al., 2019b). *Albizia myriophylla* extract have antioxidants and antimicrobial activities that show lesser aerobes and other organisms count, when incorporated in rice beer. Along with this, it also shows minimal change in the acidity, color, peroxide value, anthocyanin content with no biogenic amines during storage, hence extending the shelf-life of the alcoholic beverage (Mangang et al., 2017a). Additionally, sulfites can also act as an antioxidant that inhibits beer oxidation during storage, to contribute to the stability of the beer. Moreover, sulfite can react with the carbonyl staling compounds that affects the pleasant flavor of the beer (Guido, 2016).

**FIGURE 8:**
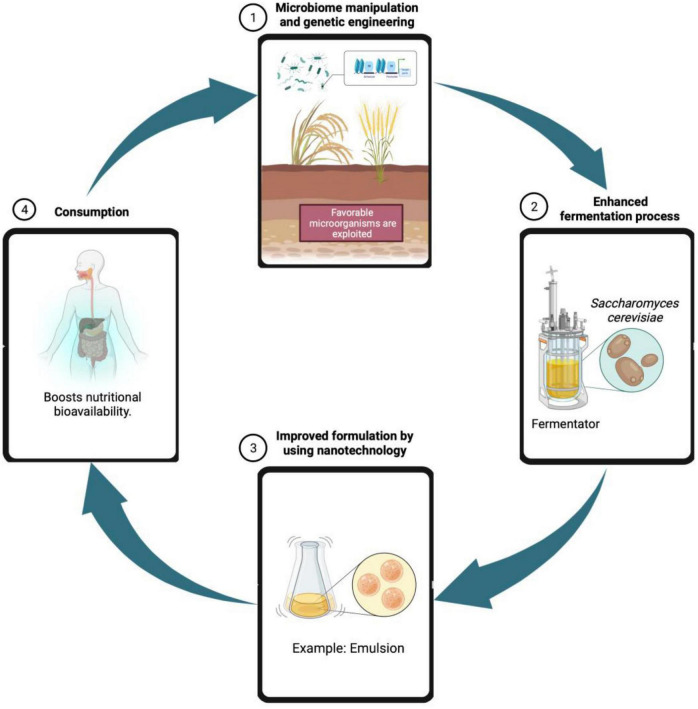
Future prospects for maximizing nutritional value of rice beer through genetic engineering, enhanced fermentation processes, and nanotechnology.

Overall, although fermented foods have been consumed since decades, their use remain prevalent till date and remains attractive, due to consumer acceptability and the beneficial characteristics present. Traditionally prepared rice beer of Assam is seen to be a chief source of nutrients as well as probiotics and shows good antioxidant activity. Traditionally prepared fermented foods and beverages native to the Northeast part of India are believed to have medicinal qualities. Although no such documentation is available, traditional culture and knowledge of the native people hold these foods in high regard where beneficial compounds in various customs and ceremonies are found. Plant parts and extracts used to produce rice beer have also been reported to have great medicinal values integrated into traditional healing remedies among the tribal communities. To produce beneficial results, a modern scientific and technological approach should be used to investigate this indigenous beverage. Further approaches could be put to develop and improve the genetic microbial strains, raw material composition and enzymatic control during fermentation, study of microbiota and synbiotic content in order to commercialize beer beverage (Figure 8).

Modernization has led to several risks in the traditional ethnic process and raw materials that compromises the good effects and quality if beverage is left over a period of time. Due to some other social and economic reasons like illegal and synthetic preparation using fertilizers and chemicals also bring loss of its good properties and compromised quality. The use of fertilizers, pesticides modern agricultural practices also compromise the rice quality hence the raw material for fermentation affects the quality of the product. Future studies should focus on product improvement through the help of various fields like medical science, biotechnology and food technology. Also, proper optimization of fermentation process, purification methods and product harvest will lead to better quality of rice beer through development of potential isolates. It also holds scope for the development of drugs and its shelf-life for commercialization.

## Conclusion

Fermentation produces probiotics, which can help restore the balance of friendly bacteria in stomach and reduce some gut problems. Probiotics have been shown to help with the symptoms of IBS, a common digestive illness. Fermented foods have been linked to a lower risk of cardiovascular disease. Probiotics may also help lower the cholesterol and blood pressure. The fermented food beverages, with their high probiotic content, can boost the immune system and lower the risk of infections. Consumption of fermented beverages resulting improvement in the gut microbiome, may have a modulatory influence on the brain and central nervous system. There is good synbiotic potential of rice-based alcoholic beverages due to their beneficial effects that can also help in its commercialization. Nevertheless, proper investigation, identification, selection and manipulation of beneficial microbes that carry out the fermentation procedure of rice beverage, can lead to a very important commercially available ethnic beverage that contains many health benefits. Biotechnological approach to improve microbial strains and manipulative selection of enriched compounds will also develop product quality further.

## Data Availability Statement

The original contributions presented in this study are included in the article/supplementary material, further inquiries can be directed to the corresponding authors.

## Author Contributions

SF, JM, MT, MS, and NF: writing – original draft. SF, JM, MT, MS, and NF: conceptualization. SF, JM, MT, MS, and NF: supervision. SF, JM, MT, MS, MB, KC, and NF: resources. SF, JM, MT, MS, SG, VS, NR, MB, KC, RN, MM, KS, PL, and NF: data curation and writing – review, and editing. All authors have read and agreed to the published version of the manuscript.

## Conflict of Interest

The authors declare that the research was conducted in the absence of any commercial or financial relationships that could be construed as a potential conflict of interest.

## Publisher’s Note

All claims expressed in this article are solely those of the authors and do not necessarily represent those of their affiliated organizations, or those of the publisher, the editors and the reviewers. Any product that may be evaluated in this article, or claim that may be made by its manufacturer, is not guaranteed or endorsed by the publisher.
